# Urinary Angiotensinogen and Progression of Chronic Kidney Disease: Results from KNOW-CKD Study

**DOI:** 10.3390/biom12091280

**Published:** 2022-09-10

**Authors:** Sang Heon Suh, Tae Ryom Oh, Hong Sang Choi, Eun Mi Yang, Chang Seong Kim, Eun Hui Bae, Seong Kwon Ma, Kook-Hwan Oh, Ji Yong Jung, Young Youl Hyun, Soo Wan Kim

**Affiliations:** 1 Department of Internal Medicine, Chonnam National University Medical School, Chonnam National University Hospital, Gwangju 64169, Korea; 2 Department of Pediatrics, Chonnam National University Medical School, Chonnam National University Hospital, Gwangju 64169, Korea; 3 Department of Internal Medicine, Seoul National University Hospital, Seoul 03080, Korea; 4 Department of Internal Medicine, Gachon University of Medicine and Science, Incheon 21565, Korea; 5 Department of Internal Medicine, Kangbuk Samsung Hospital, Sungkyunkwan University School of Medicine, Seoul 03181, Korea

**Keywords:** angiotensinogen, biomarker, chronic kidney disease, end-stage renal disease, estimated glomerular filtration rate

## Abstract

The prognostic value of urinary angiotensinogen (UAGT) in patients with chronic kidney disease (CKD) has not been completely evaluated, although the association of UAGT with renal outcomes has been suggested in specific subsets of CKD. In the present study, to investigate the association of UAGT with renal outcomes in patients with non-dialysis CKD irrespective of the primary cause, a total of 1688 subjects from the Korean Cohort Study for Outcomes in Patients With Chronic Kidney Disease (KNOW-CKD) were prospectively analyzed. The subjects were divided into the quintile by UAGT to urine creatinine ratio (UAGT/Cr) level. The primary outcomes of interest were composite renal event, which included decline in kidney function and onset of end-stage renal disease during follow-up periods. The median follow-up duration was 6.257 years. Cox regression model analysis unveiled that the risk of composite renal event was significantly higher in the fifth quintile (adjusted hazard ratio 1.528, 95% confidence interval 1.156 to 2.021) compared to that of the first quartile. The association between high UAGT/Cr level and adverse renal outcome remained consistent in sensitivity analyses, including the analysis of the cause-specific hazard model. Subgroup analyses revealed that the association of UAGT level with renal outcomes is modified by certain clinical contexts, such as BMI and albuminuria. In conclusion, high UAGT level is associated with adverse renal outcomes in patients with non-dialysis CKD. Further studies are warranted to elaborate and expand the predictive role of UAGT as a biomarker for renal outcomes in CKD.

## 1. Introduction

Angiotensinogen (AGT) is a peptide molecule with a molecular weight of 53- to 75-kDa depending on the extent of glycosylation [1], and is the only known substrate for renin that is the rate-limiting enzyme of the renin–angiotensin system (RAS) [2]. The changes in AGT level, along with renin activity, control the overall activity of RAS [2,3], where renin enzymatically processes AGT to angiotensin I (AngI), which is further cleaved by angiotensin-converting enzyme (ACE) to form AngII. AngII, the major effector molecule of classic RAS, binds to its cognate G-protein-coupled receptor, AngII type 1 receptor (AT1R), leading to water and salt retention, vasoconstriction, and proliferative, proinflammatory, and profibrotic processes [4]. The inhibition of RAS with ACE inhibitors (ACEi) and AngII receptor blockers (ARB) is a cornerstone in the management of patients with chronic kidney disease (CKD), presenting additional kidney protection beyond their blood pressure (BP)-lowering effect [5,6].

The tissue-specific regulation of RAS is independent of the circulating level of AngII in various organs, such as the heart and kidney [7,8]. In contrast to the other organs, the kidney has all the components of RAS [3], and local tissue concentrations of AngII in the kidney are far greater than can be derived from circulating AngII concentrations [9,10,11,12]. As the upregulation of intrarenal RAS components correlates with fibrotic tissue injury in progressive kidney diseases [13,14,15], it has been previously proposed that urinary AngII could be a biomarker of intrarenal RAS activity [16]; albeit, it is currently difficult to measure the precise level of AngII in patients. Instead, it has been reported that urinary AGT (UAGT) level correlates with intrarenal AGT and AngII levels in rodent models of hypertensive nephropathy [17,18,19]. A translational study also reported that UAGT may be a marker of intrarenal AngII activity in patients with CKD [20]. Accordingly, it has been reported that UAGT is associated with adverse renal outcomes in specific subsets of patients with CKD, such as those with type 2 diabetes mellitus (DM) [21,22,23] and polycystic kidney disease (PKD) [24,25]. Yet, the prognostic value of UAGT in patients with CKD of various etiologies has not been completely evaluated. 

In the present study, we aimed to investigate the association of UAGT with renal outcomes in patients with non-dialysis CKD, irrespective of the primary cause. A series of sensitivity analyses are included to validate our findings. Finally, we conducted subgroup analyses to examine whether the association between UAGT level and renal outcomes might be modified clinical contexts.

## 2. Materials and Methods

### 2.1. Study Design

**Figure 1:**
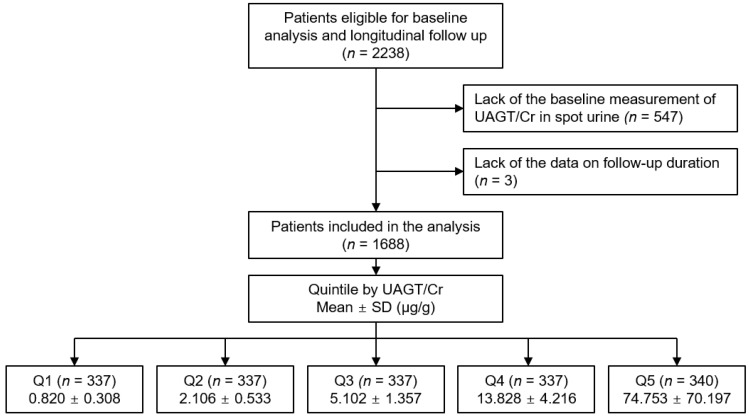
Flow diagram of the study participants. Abbreviations: Q1, 1st quintile; Q2, 2nd quintile; Q3, 3rd quintile; Q4, 4th quintile; Q5, 5th quintile; UAGT/Cr, urinary angiotensinogen-to-creatinine ratio.

The Korean Cohort Study for Outcomes in Patients With Chronic Kidney Disease (KNOW-CKD) is a nationwide prospective cohort study involving 9 tertiary-care general hospitals in Korea (NCT01630486 at http://www.clinicaltrials.gov, accessed on 1 September 2022) [26]. Korean patients, aged between 20 and 75 years, with CKD from stage 1 to pre-dialysis stage 5, who voluntarily provided informed consent were enrolled from 2011 to 2016. The study was conducted in accordance with the principles of the Declaration of Helsinki. The study protocol was approved by the institutional review boards of participating centers, including at Seoul National University Hospital, Yonsei University Severance Hospital, Kangbuk Samsung Medical Center, Seoul St. Mary’s Hospital, Gil Hospital, Eulji General Hospital, Chonnam National University Hospital, and Busan Paik Hospital. All participants had been under close observation, and participants who experienced study outcomes were reported by each participating center. Among 2238 who were longitudinally followed up, excluding those lacking the baseline measurement of AGT or creatinine (Cr) in spot urine, and those lacking the data on follow-up duration, a total of 1688 subjects were finally included for the analyses (Figure 1). The study observation period ended on 31 March 2021. The median follow-up duration was 6.257 years.

### 2.2. Data Collection from Participants

Demographic information was collected from all eligible participants, including age, gender, comorbid conditions, primary renal disease, smoking history, and medication history (ACEi/ARBs, diuretics, number of antihypertensive drugs, and statins). Trained staff members measured the height and weight of study participants. Body mass index (BMI) was calculated as weight divided by the height squared. Systolic and diastolic blood pressures (SBP and DBP) were measured by an electronic sphygmomanometer after seated rest for 5 min. Venous samples were collected following overnight fasting, to determine hemoglobin, albumin, total cholesterol, LDL-C, HDL-C, TG, fasting glucose, high-sensitivity C-reactive protein (hs-CRP), 25-hydroxyvitamin D (25(OH) vitamin D), and creatinine (Cr) levels at the baseline. eGFR was calculated using the Chronic Kidney Disease Epidemiology Collaboration equation [27]. CKD stages were determined by the Kidney Disease Improving Global Outcomes guidelines [28]. Urine albumin-to-Cr ratio (ACR) was measured in random, preferably second-voided, spot urine samples.

### 2.3. Determination of Urinary Angiotensinogen-to-Creatinine Ratio (UAGT/Cr)

Spot urine samples were collected from the participants to measure UAGT levels. The urine samples were centrifuged at 1500 rpm for 10 min at 4 °C. The urinary supernatants were pooled, and the UAGT concentrations were measured using human AGT ELISA kits (IBL, Takasaki, Japan), where intra-assay and inter-assay coefficients of variation were 4.4% and 4.3%, respectively [29]. UAGT level was normalized to urinary Cr contents, as previously described [30].

### 2.4. Exposure and Study Outcome

The exposure of primary interest was UAGT/Cr level, which was used as a categorical variable. The subjects were divided into the quintile (Q1, Q2, Q3, Q4 and Q5) by UAGT/Cr level (Figure 1). The primary outcomes of interest were composite renal events. Composite renal events included decline in kidney function (the first occurrence of >50% decline in eGFR or doubling of serum Cr from the baseline) and onset of end-stage renal disease (ESRD, initiation of dialysis or kidney transplantation) during follow-up periods. The secondary outcomes were decline in kidney function and onset of ESRD.

### 2.5. Statistical Analysis

Continuous variables were expressed as mean ± standard deviation or median [interquartile range]. Categorical variables were expressed as number of participants and percentage. Normality of distribution was ascertained by the Kolmogorov–Smirnov test. To compare the baseline characteristics by UAGT/Cr, one-way analysis of variance and χ^2^ test were used for continuous and categorical variates, respectively. Cumulative incidences of composite renal events, decline in kidney function and onset of ESRD were estimated using Kaplan–Meier analyses, and were compared using log-rank test. The participants with any missing data were excluded for further analyses in the primary analysis. To evaluate the association between UAGT/Cr level and study outcomes, Cox proportional hazard regression models were analyzed. Patients lost to follow-up were censored at the date of the last visit. Models were constructed after adjusting for the following variables. Model 1 represents crude hazard ratios (HRs). Model 2 was adjusted for age, sex, age-adjusted Charlson comorbidity index, primary renal disease, current smoking status, medication (ACEi/ARB, diuretics, number of antihypertensive drugs, or statins), BMI, SBP, and DBP. Model 3 was further adjusted for hemoglobin, albumin, fasting glucose, total cholesterol, high-density lipoprotein cholesterol (HDL-C), low-density lipoprotein cholesterol (LDL-C), triglycerides (TG), 25(OH) vitamin D, and hs-CRP. Model 4 was finally adjusted for serum Cr level and spot urine ACR. The results of Cox proportional hazard models were presented as HRs and 95% confidence intervals (CIs). Restricted cubic splines were used to visualize the association between UAGT/Cr level as a continuous variable and HRs for study outcomes. To validate our findings, we performed sensitivity analyses. First, we excluded the subjects with eGFR ≥ 90 mL/min/1.73 m^2^ (CKD stage 1), because the subjects with eGFR ≥ 90 mL/min/1.73 m^2^ are considered close to normal kidney function, and may not represent the CKD population well. Second, we excluded the subjects with eGFR < 15 mL/min/1.73 m^2^ (CKD stage 5), because the subjects with eGFR < 15 mL/min/1.73 m^2^ are relatively small in number, and may exaggerate the association between serum TG level and study outcomes due to far-advanced CKD. Third, we excluded the subjects with DM or PKD as a primary cause of CKD, as the association of UAGT/Cr with renal outcomes among the subjects with DM or PKD was previously demonstrated [21,22,23,24,25]. Fourth, we assessed cause-specific HRs for the primary study outcome by UAGT/Cr levels, where death before reaching the composite renal event was considered a competing risk and treated as censoring. Fifth, we replaced the missing values in primary analyses by a multiple imputation, and further conducted Cox regression analyses. Sixth, the participants were divided into the quartile by UAGT/Cr level, instead of the quintile. Lastly, the co-variate ‘serum creatinine level’ was replaced with ‘eGFR’ to estimate HR and 95% CI. To examine whether the association of UAGT/Cr level with study outcomes is modified by certain clinical contexts, we conducted pre-specified subgroup analyses. Subgroups were defined by age (<60 versus (vs.) ≥60 years), sex (male vs. female), BMI (<23 vs. ≥23 kg/m^2^), eGFR (<45 vs. ≥45 mL/min/1.73 m^2^), and spot urine ACR (<300 vs. ≥300 mg/g). Two-sided *p* values < 0.05 were considered statistically significant. Statistical analysis was performed using SPSS for Windows v22.0 (IBM Corp., Armonk, NY, USA) and R (v4.1.1; R project for Statistical Computing, Vienna, Austria).

## 3. Results

### 3.1. Baseline Characteristics

To describe the baseline characteristics, the study participants were divided into the quintile by UAGT/Cr level (Table 1). The mean age of the participants was higher in the subjects in the fifth quartile (Q5) than those in the first (Q1), second (Q2), third (Q3) and fourth (Q4) quintile. The proportion of male participants was the highest in Q5. The proportion of the participants with age-adjusted Charlson comorbidity index 0–3 was lowest in Q5, whereas those with age-adjusted Charlson comorbidity index 6–7 was also most frequently observed in Q5. The history of DM was most frequent in Q5, whereas the prevalence of PKD was lowest Q5. The frequency of current smokers was highest in Q1. The use of diuretics and antihypertensive drugs no less than three classes was most prevalent in Q5. Hemoglobin and albumin levels were lowest in Q5, while HDL-C levels were lowest in Q1. TG and fasting glucose levels were highest in Q1. 25(OH) vitamin D level was significantly lower in Q5. Spot urine ACR and serum creatinine levels were significantly higher in Q5. Accordingly, eGFR was significantly lower in Q5, while the frequency of advanced CKD was relatively higher in Q5.

### 3.2. Association of UAGT/Cr Level with Renal Outcomes in Patients with Non-Dialysis CKD

**Figure 2:**
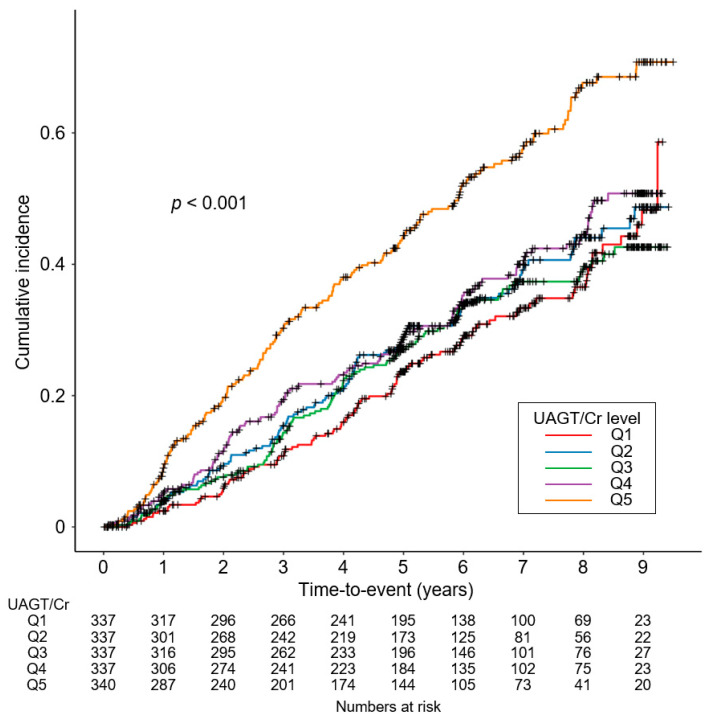
Kaplan–Meier survival curve for cumulative incidence of composite renal events by UAGT/Cr. *p* value by log-rank test. Abbreviations: Q1, 1st quintile; Q2, 2nd quintile; Q3, 3rd quintile; Q4, 4th quintile; Q5, 5th quintile; UAGT/Cr, urinary angiotensinogen-to-creatinine ratio.

**Figure 3:**
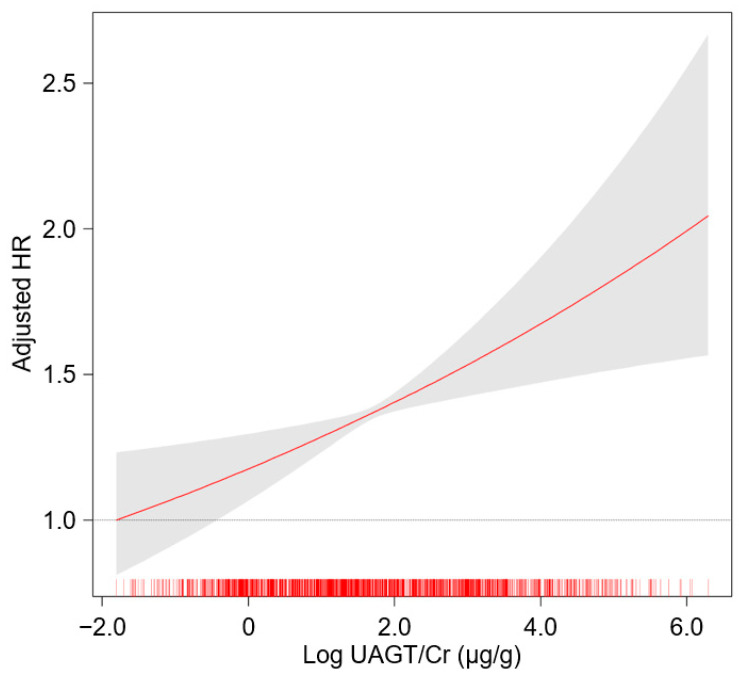
Restricted cubic spline of UAGT/Cr on composite renal event. Adjusted HR of UAGT/Cr as a continuous variable for composite renal event is depicted. The model was adjusted for age, sex, age-adjusted Charlson comorbidity index, primary renal disease, current smoking, medications (ACEi/ARBs, diuretics, number of antihypertensive drugs, statins), BMI, SBP, DBP, hemoglobin, albumin, fasting glucose, total cholesterol, LDL-C, HDL-C, TG, 25(OH) vitamin D, hs-CRP, serum creatinine and spot urine ACR. Abbreviations: HR, hazard ratio; UAGT/Cr, urinary angiotensinogen-to-creatinine ratio.

To unveil the cumulative incidences of composite renal event (Figure 2), decline in kidney function (Figure S1) and onset of ESRD (Figure S2), Kaplan–Meier curves were analyzed. The risks of composite renal event (*p* < 0.001, by log-rank test), decline in kidney function (*p* = 0.025, by log-rank test) and onset of ESRD (*p* < 0.001, by log-rank test) were significantly different by UAGT/Cr, with the highest risk of the events in Q5. To define the independent association of UAGT/Cr level with study outcomes, Cox regression models were analyzed. The risk of a composite renal event was significantly higher in Q5 (adjusted HR 1.528, 95% CI 1.156 to 2.021) compared to that of Q1 (Table 2), suggesting that high UAGT level is associated with adverse renal outcome. Although the risk of decline in kidney function was not significantly different according to UAGT/Cr levels (Table S1), the risk of onset of ESRD (adjusted HR 1.444, 95% CI 1.050 to 1.987) was significantly higher in Q5 compared to that of Q1 (Table S2). Restricted cubic splines visualized stringent linear correlations of UAGT/Cr level with the risks of composite renal event (Figure 3). As in the Cox regression analysis, no significant linear correlation was observed between UAGT/Cr level and the risk of decline in kidney function (Figure S3), whereas UAGT/Cr level was positively correlated with the risk of onset of ESRD (Figure S4).

### 3.3. Sensitivity Analyses

To validate the findings, we performed sensitivity analyses. After excluding the subjects with eGFR ≥ 90 mL/min/1.73 m^2^ (adjusted HR 1.449, 95% CI 1.087 to 1.932), or after excluding the subjects with eGFR < 15 mL/min/1.73 m^2^ (adjusted HR 1.444, 95% CI 1.069 to 1.951), the risk of composite renal event remained significantly higher in Q5, compared to that of Q1 (Tables S3 and S4). The association between UAGT/Cr level and composite renal outcome was still robust (adjusted HR 1.901, 95% CI 1.335 to 2.707) even after excluding the subjects with DM (Table S5). In the analysis excluding the subjects with PKD (Table S6), the HR for composite renal outcome by UAGT/Cr level was still significant (adjusted HR 1.363, 95% CI 1.009 to 1.841). Next, we analyzed a cause-specific hazard model for the primary study outcome by UAGT/Cr levels, where the risk of composite renal event was robustly higher in Q5 (adjusted HR 1.528, 95% CI 1.144 to 2.041) compared to that of Q1 (Table 3). After replacing the missing values by multiple imputation, the risk of composite renal event remained robustly higher in Q5 (adjusted HR 1.447, 95% CI 1.111 to 1.886) compared to that of Q1 (Table 4). Even when the participants were divided into the quartile by UAGT/Cr level, instead of the quintile, the highest quartile revealed significantly increased risk of composite renal event (adjusted HR 1.385, 95% CI 1.078 to 1.778, *p* = 0.011) (Table S7). Finally, after replacing the co-variate ‘serum creatinine level’ with ‘eGFR’, the risk of composite renal event was still higher in Q5 (adjusted HR 1.427, 95% CI 1.078 to 1.888) compared to that of Q1 (Table S8).

### 3.4. Subgroup Analyses

To examine whether the association of UAGT/Cr level with the risk of composite renal event is modified by certain clinical contexts, we conducted pre-specified subgroup analyses. The association between UAGT/Cr level and composite renal event was significantly more prominent in the subjects with BMI < 23 kg/m^2^ (*p* for interaction <0.001) and spot urine ACR ≥ 300 mg/g (*p* for interaction = 0.043) (Table 5).

## 4. Discussion

In the present study, we demonstrated that high UAGT level is associated with adverse renal outcome in patients with non-dialysis CKD. In particular, high UAGT level was associated with increased risk of onset of ESRD. Our finding is robust, because we demonstrated consistent results in a series of sensitivity analyses, including the analysis of cause-specific hazard models, and analysis with multiple imputation. We also observed that the association was modified by certain clinical contexts, such as BMI and albuminuria.

Despite the role of UAGT as a surrogate of intrarenal RAS activity [17,18,19,20] and the clinical relevance of intrarenal RAS with renal prognosis in patients with CKD [13,14,15], direct evidence to support the association of UAGT with renal outcomes has been surprisingly lacking. The predictive value of UAGT in kidney outcomes has been partially reported in patients with DM [21,22,23] or PKD [24,25]. Ishigaki et al. reported that elevated baseline UAGT level can predict renal dysfunction in patients with CKD irrespective of the primary cause, whereas the study analyzed only 62 patients with a relatively short duration of follow-up period (i.e., one year) [31]. In contrast, we here included a total of 1688 subjects with CKD with various etiologies, with median follow-up duration of 6.257 years. Moreover, the present study included sensitivity analyses excluding the subjects with DM (Table S5) or PKD (Table S6), to prevent the major finding of the study being primarily driven by those subpopulations, where the association between UAGT/Cr and the risk of composite renal event remained robust. In this regard, the current study presented solid evidence that, among patients with non-dialysis CKD regardless of the primary cause, high UAGT level is associated with adverse renal outcomes.

Although the association of UAGT at the baseline with renal outcomes has been demonstrated in the current study, we believe that the potential role of UAGT in the prediction of kidney outcomes should be further elaborated and expanded. First, it remains still elusive that the changes in UAGT (i.e., elevation or reduction in UAGT level during the follow-up period) predicts an improvement in or deterioration of renal prognosis in patients with CKD. Second, the predictive value of UAGT level after initiation of ACEi/ARB in the kidney outcomes should be clarified. Although ACEi/ARB is the fundamental axis of the management in patients with CKD, and most of the patients with CKD are treated with ACEi/ARB, not all the cases are successful. In this context, it is hypothesized that the ‘residual’ activity of intrarenal RAS after initiation of ACEi/ARB treatment would be reflected in UAGT level, and that UAGT level after initiation of ACEi/ARB may more precisely predict the kidney outcomes. Third, we suggest that UAGT level after the initiation of ACEi/ARB may help to define a specific subpopulation to be treated with dual pharmacotherapy of ACEi and ARB. Currently, no evidence supports the beneficial effect of combination treatment of ACEi and ARB in patients with CKD [32]. Yet, we suppose that dual treatment may be considered in the patients with high UAGT level even after initiation of monotherapy, as UAGT level is associated with the therapeutic response to ACEi/ARB [33]. Further studies, therefore, are warranted to determine the predictive role of UAGT after initiation of ACEi/ARB in the kidney outcomes.

There are a number of limitations to be acknowledged in the present study. First, we cannot determine the casual relation between high UAGT and CKD progression, because of the observational nature of the present study. It could, however, be postulated that high UAGT predicts CKD progression, based on the evidence indicating the role of UAGT as a surrogate of intrarenal RAS activity [17,18,19,20] and the clinical relevance of intrarenal RAS with renal prognosis in patients with CKD [13,14,15]. Second, all the variables were measured once at the baseline. However, the previous observational studies [21,22,23,24,25,31], which share the same limitation, reported similar results that are largely concordant with ours. We assume that, hence, the single measurement of the variables at the baseline does not interfere with the overall significance of the results presented in the current study. Third, as this cohort study enrolled only ethnic Koreans, a precaution is required to extrapolate the data to other populations. It should be noted that, however, a similar result was reported by a study conducted in Japan [31]. Fourth, the use of a class of potent renoprotective agents, sodium-glucose cotransporter-2 inhibitors (SGLT2 inhibitors), were not included as a co-variate in the analysis model, because only one participant was being treated with an SGLT2 inhibitor at the beginning of the study. This could be attributed to the time point at which the KNOW-CKD launched in 2011, when SGLT2 inhibitors were not introduced into routine clinical practice.

In conclusion, we report that high UAGT level is associated with adverse renal outcomes in patients with non-dialysis CKD. In particular, a high UAGT level is associated with increased risk of onset of ESRD. We also report that the association is modified by certain clinical contexts, such as BMI and albuminuria. Further studies are warranted to elaborate and expand the predictive role of UAGT as a biomarker for renal outcomes in CKD.

## Figures and Tables

**Table 1:** Baseline characteristics of study participants by UAGT/Cr.

			UAGT/Cr			
	Q1	Q2	Q3	Q4	Q5	*p*-Value
Age (year)	53.086 ± 12.525	52.632 ± 12.332	52.855 ± 12.797	51.804 ± 12.397	55.447 ± 11.665	0.001
Male	253 (75.1)	217 (64.4)	187 (55.5)	188 (55.8)	164 (48.2)	<0.001
Age-adjusted CCI						0.001
0–3	181 (53.7)	189 (56.1)	202 (59.9)	199 (59.1)	146 (42.9)	
4–5	100 (29.7)	86 (25.5)	79 (23.4)	88 (26.1)	108 (31.8)	
6–7	48 (14.2)	50 (14.8)	49 (14.5)	38 (11.3)	73 (21.5)	
≥8	8 (2.4)	12 (3.6)	7 (2.1)	12 (3.6)	13 (3.8)	
Primary renal disease						<0.001
DM	91 (27.0)	71 (21.1)	69 (20.5)	69 (20.5)	112 (33.0)	
HTN	71 (21.1)	76 (22.6)	66 (19.6)	54 (16.0)	48 (14.2)	
GN	106 (31.5)	92 (27.3)	103 (30.6)	133 (39.5)	111 (32.7)	
TID	1 (0.3)	2 (0.6)	2 (0.6)	4 (1.2)	4 (1.2)	
PKD	49 (14.5)	76 (22.6)	74 (22.0)	56 (16.6)	40 (11.8)	
Others	19 (5.6)	20 (5.9)	23 (6.8)	21 (6.2)	24 (7.1)	
Current smoker	70 (20.8)	53 (15.7)	45 (13.4)	52 (15.4)	52 (15.4)	0.108
Medication						
ACEi/ARBs	287 (85.2)	283 (84.0)	288 (85.5)	289 (85.8)	301 (88.8)	0.469
Diuretics	97 (28.8)	81 (24.0)	89 (26.4)	99 (29.4)	132 (38.9)	<0.001
Anti-HTN drugs ≥ 3	97 (28.8)	93 (27.6)	83 (24.6)	89 (26.4)	119 (35.1)	0.031
Statins	172 (51.0)	168 (49.9)	159 (47.2)	176 (52.2)	182 (53.7)	0.509
BMI (kg/m^2^)	24.749 ± 3.143	24.553 ± 3.334	24.739 ± 3.486	24.372 ± 3.442	24.558 ± 3.640	0.583
SBP (mmHg)	126.561 ± 15.407	127.484 ± 16.103	127.092 ± 15.203	128.843 ± 16.666	129.817 ± 17.124	0.062
DBP (mmHg)	76.279 ± 11.229	77.736 ± 10.771	77.178 ± 11.121	78.134 ± 11.629	77.354 ± 11.122	0.279
Laboratory findings						
Hemoglobin (g/dL)	13.250 ± 2.145	13.151 ± 1.977	12.939 ± 1.919	12.897 ± 1.943	12.255 ± 1.937	<0.001
Albumin (g/dL)	4.257 ± 0.407	4.233 ± 0.405	4.214 ± 0.352	4.153 ± 0.408	4.054 ± 0.508	<0.001
Total cholesterol (mg/dL)	173.131 ± 38.905	174.804 ± 40.904	176.167 ± 35.360	175.716 ± 38.823	179.629 ± 40.081	0.302
HDL-C (mg/dL)	47.335 ± 16.757	49.248 ± 13.548	50.880 ± 15.383	50.921 ± 15.237	49.481 ± 16.009	0.026
LDL-C (mg/dL)	95.682 ± 31.445	98.132 ± 32.606	98.056 ± 28.084	98.366 ± 30.841	99.672 ± 32.780	0.604
TG (mg/dL)	176.181 ± 115.216	149.664 ± 103.085	152.788 ± 94.411	151.128 ± 91.875	168.695 ± 101.411	0.002
Fasting glucose (mg/dL)	115.491 ± 42.304	108.606 ± 37.814	107.364 ± 35.074	107.826 ± 34.445	111.815 ± 40.850	0.043
25(OH) Vitamin D (ng/mL)	18.137 ± 8.147	18.019 ± 7.183	17.817 ± 7.697	18.433 ± 7.990	16.653 ± 8.124	0.046
hs-CRP (mg/dL)	0.600 [0.278, 1.502]	0.500 [0.140, 1.400]	0.600 [0.200, 1.600]	0.600 [0.253, 1.600]	0.600 [0.220, 1.900]	0.700
Spot urine ACR (mg/g)	254.158 [42.897, 1485.141]	244.308 [29.798, 767.609]	256.422 [38.550, 897.591]	458.005 [141.671, 1329.344]	747.315 [267.141, 4207.912]	<0.001
Creatinine (mg/dL)	1.699 ± 1.002	1.710 ± 1.171	1.656 ± 1.030	1.784 ± 1.211	2.139 ± 1.267	<0.001
eGFR (mL/min/1.73 m^2^)	54.075 ± 30.130	54.017 ± 30.369	55.203 ± 32.756	52.641 ± 31.247	40.943 ± 27.285	<0.001
CKD stages						<0.001
Stage 1	57 (16.9)	66 (19.6)	75 (22.3)	66 (19.6)	32 (9.4)	
Stage 2	75 (22.3)	73 (21.7)	61 (18.1)	66 (19.6)	49 (14.4)	
Stage 3a	65 (19.3)	47 (13.9)	49 (14.5)	51 (15.1)	49 (14.4)	
Stage 3b	68 (20.2)	72 (21.4)	67 (19.9)	71 (21.1)	71 (20.9)	
Stage 4	61 (18.1)	66 (19.6)	72 (21.4)	61 (18.1)	97 (28.5)	
Stage 5	11 (3.3)	13 (3.9)	13 (3.9)	22 (6.5)	42 (12.4)	

Values for categorical variables are given as number (percentage); values for continuous variables, as mean ± standard deviation or median (interquartile range). Abbreviations: ACEi, angiotensin-converting enzyme inhibitor; ACR, albumin-to-creatinine ratio; ARB, angiotensin receptor blocker; BMI, body mass index; CCI, Charlson comorbidity index; CKD, chronic kidney disease; DBP, diastolic blood pressure; DM, diabetes mellitus; eGFR, estimated glomerular filtration rate; GN, glomerulonephritis; HDL-C, high-density lipoprotein cholesterol; hs-CRP, high-sensitivity C-reactive protein; HTN, hypertension; LDC-C, low-density lipoprotein cholesterol; PKD, polycystic kidney disease; SBP, systolic blood pressure; TG, triglyceride; TID, tubulointerstitial disease; Q1, 1st quintile; Q2, 2nd quintile, Q3, 3rd quintile; Q4, 4th quintile; Q5, 5th quintile; UAGT/Cr, urinary angiotensinogen-to-creatinine ratio.

**Table 2:** HRs for composite renal event by UAGT/Cr level.

	UAGT/Cr	Events, n (%)	Model 1		Model 2		Model 3		Model 4	
			HR (95% CIs)	*p*-Value	HR (95% CIs)	*p*-Value	HR (95% CIs)	*p*-Value	HR (95% CIs)	*p*-Value
**Composite Renal Event**	**Q1**	**104 (30.9)**	**Reference**		**Reference**		**Reference**		**Reference**	
	Q2	111 (32.9)	1.178 (0.884, 1.571)	0.263	1.431 (1.092, 1.876)	0.009	1.472 (1.098, 1.974)	0.010	1.160 (0.849, 1.585)	0.350
	Q3	113 (33.5)	1.097 (0.827, 1.456)	0.519	1.419 (1.080, 1.864)	0.012	1.461 (1.092, 1.956)	0.011	1.396 (1.042, 1.870)	0.025
	Q4	125 (37.1)	1.396 (1.062, 1.836)	0.017	1.472 (1.128, 1.921)	0.004	1.560 (1.175, 2.072)	0.002	1.324 (0.995, 1.763)	0.054
	Q5	179 (52.6)	2.341 (1.809, 3.030	<0.001	2.027 (1.581, 2.598)	<0.001	1.968 (1.499, 2.584)	<0.001	1.528 (1.156, 2.021)	0.003

Model 1, unadjusted model. Model 2, model 1 + adjusted for age, sex, age-adjusted Charlson comorbidity index, primary renal disease, current smoking, medication (ACEi/ARBs, diuretics, number of antihypertensive drugs, statins), BMI, SBP and DBP. Model 3, model 2 + adjusted for hemoglobin, albumin, total cholesterol, LDL-C, HDL-C, TG, fasting glucose, 25(OH) vitamin D, and hs-CRP. Model 4, model 3 + serum creatinine and spot urine ACR. Abbreviations: CI, confidence interval; HR, hazard ratio; Q1, 1st quintile; Q2, 2nd quintile; Q3, 3rd quintile; Q4, 4th quintile; Q5, 5th quintile; UAGT/Cr, urinary angiotensinogen-to-creatinine ratio.

**Table 3:** Cause-specific HRs for composite renal event by UAGT/Cr level.

	UAGT/Cr	Model 1		Model 2		Model 3		Model 4	
		HR (95% CIs)	*p*-Value	HR (95% CIs)	*p*-Value	HR (95% CIs)	*p*-Value	HR (95% CIs)	*p*-Value
Composite Renal Event	Q1	Reference		Reference		Reference		Reference	
	Q2	1.170 (0.900, 1.521)	0.240	1.431 (1.090, 1.879)	0.010	1.472 (1.085, 1.997)	0.013	1.160 (0.842, 1.598)	0.362
	Q3	1.060 (0.815, 1.378)	0.666	1.418 (1.070, 1.882)	0.015	1.461 (1.079, 1.978)	0.014	1.396 (1.044, 1.866)	0.024
	Q4	1.255 (0.972, 1.621)	0.081	1.472 (1.124, 1.929)	0.005	1.561 (1.147, 2.123)	0.004	1.324 (0.970, 1.807)	0.077
	Q5	2.177 (1.717, 2.760)	<0.001	2.027 (1.568, 2.620)	<0.001	1.968 (1.462, 2.650)	<0.001	1.528 (1.144, 2.041)	0.004

Model 1, unadjusted model. Model 2, model 1 + adjusted for age, sex, age-adjusted Charlson comorbidity index, primary renal disease, current smoking, medication (ACEi/ARBs, diuretics, number of antihypertensive drugs, statins), BMI, SBP and DBP. Model 3, model 2 + adjusted for hemoglobin, albumin, total cholesterol, LDL-C, HDL-C, TG, fasting glucose, 25(OH) vitamin D, and hs-CRP. Model 4, model 3 + serum creatinine and spot urine ACR. Abbreviations: CI, confidence interval; HR, hazard ratio; Q1, 1st quintile; Q2, 2nd quintile; Q3, 3rd quintile; Q4, 4th quintile; Q5, 5th quintile; UAGT/Cr, urinary angiotensinogen-to-creatinine ratio.

**Table 4:** HRs for composite renal event by UAGT/Cr level using a multiple imputation.

	UAGT/Cr	Model 1		Model 2		Model 3		Model 4	
		HR (95% CIs)	*p*-Value	HR (95% CIs)	*p*-Value	HR (95% CIs)	*p*-Value	HR (95% CIs)	*p*-Value
Composite Renal Event	Q1	Reference		Reference		Reference		Reference	
	Q2	1.170 (0.895, 1.529)	0.251	1.436 (1.096, 1.882)	0.009	1.428 (1.084, 1.881)	0.012	1.131 (0.846, 1.511)	0.406
	Q3	1.060 (0.812, 1.383)	0.670	1.426 (1.086, 1.872)	0.011	1.391 (1.054, 1.836)	0.020	1.323 (1.002, 1.746)	0.049
	Q4	1.255 (0.968, 1.628)	0.087	1.461 (1.119, 1.9070)	0.005	1.367 (1.043, 1.794)	0.024	1.176 (0.895, 1.545)	0.246
	Q5	2.176 (1.709, 2.772)	<0.001	2.020 (1.575, 2.589)	<0.001	1.831 (1.421, 2.360)	<0.001	1.447 (1.111, 1.886)	0.006

Model 1, unadjusted model. Model 2, model 1 + adjusted for age, sex, age-adjusted Charlson comorbidity index, primary renal disease, current smoking, medication (ACEi/ARBs, diuretics, number of antihypertensive drugs, statins), BMI, SBP and DBP. Model 3, model 2 + adjusted for hemoglobin, albumin, total cholesterol, LDL-C, HDL-C, TG, fasting glucose, 25(OH) vitamin D, and hs-CRP. Model 4, model 3 + serum creatinine and spot urine ACR. Abbreviations: CI, confidence interval; HR, hazard ratio; Q1, 1st quintile; Q2, 2nd quintile; Q3, 3rd quintile; Q4, 4th quintile; Q5, 5th quintile; UAGT/Cr, urinary angiotensinogen-to-creatinine ratio.

**Table 5:** HRs for composite renal events by UAGT/Cr in various subgroups.

	AACS	Events, n (%)	Unadjusted HR (95% CIs)	*p* for Interaction	Adjusted HR (95% CIs)	*p* for Interaction
**Age < 60 years**	Q1	70 (32.1)	Reference	0.125	Reference	0.118
	Q2	71 (31.3)	1.040 (0.748, 1.447)		1.076 (0.730, 1.587)	
	Q3	66 (28.8)	0.835 (0.596, 1.169)		1.239 (0.851, 1.802)	
	Q4	88 (37.8)	1.196 (0.874. 1.637)		1.285 (0.905, 1.825)	
	Q5	112 (54.1)	2.027 (1.503, 2.732)		1.615 (1.130, 2.309)	
**Age ≥ 60 years**	Q1	34 (28.6)	Reference		Reference	
	Q2	40 (36.4)	1.530 (0.965, 2.427)		1.638 (0.947, 2.831)	
	Q3	47 (43.5)	1.705 (1.092, 2.661)		1.381 (0.829, 2.300)	
	Q4	37 (35.6)	1.406 (0.879, 2.249)		1.064 (0.624, 1.815)	
	Q5	67 (50.4)	2.573 (1.695, 3.905)		1.515 (0.932, 2.463)	
**Male**	Q1	77 (30.4)	Reference	0.109	Reference	0.157
	Q2	72 (33.2)	1.148 (0.833, 1.584)		1.165 (0.799, 1.699)	
	Q3	60 (32.1)	1.039 (0.741, 1.456)		1.506 (1.041, 2.177)	
	Q4	78 (41.5)	1.478 (1.079, 2.026)		1.436 (1.012, 2.040)	
	Q5	77 (47.0)	1.892 (1.379, 2.595)		1.368 (0.933, 2.006)	
**Female**	Q1	27 (32.1)	Reference		Reference	
	Q2	39 (32.5)	1.234 (0.755, 2.017)		1.436 (0.817, 2.524)	
	Q3	53 (35.3)	1.105 (0.695, 1.756)		1.399 (0.822, 2.381)	
	Q4	47 (31.5)	1.023 (0.637, 1.643)		0.837 (0.487, 1.441)	
	Q5	102 (58.0)	2.512 (1.643, 3.841)		1.396 (0.850, 2.293)	
**BMI < 23 kg/m^2^**	Q1	28 (28.9)	Reference	0.122	Reference	<0.001
	Q2	40 (38.8)	1.603 (0.989, 2.598)		3.136 (1.730, 5.685)	
	Q3	25 (25.0)	0.848 (0.494, 1.455)		1.913 (1.003, 3.648)	
	Q4	46 (38.7)	1.488 (0.930, 2.380)		1.562 (0.866, 2.815)	
	Q5	64 (56.1)	2.440 (1.565, 3.806)		3.030 (1.694, 5.425)	
**BMI ≥ 23 kg/m^2^**	Q1	76 (31.7)	Reference		Reference	
	Q2	71 (30.3)	1.012 (0.733, 1.399)		0.810 (0.555, 1.183)	
	Q3	88 (37.1)	1.139 (0.838, 1.549)		1.234 (0.880, 1.730)	
	Q4	79 (36.2)	1.516 (0.843, 1.584)		1.099 (0.777, 1.554)	
	Q5	115 (50.9)	2.068 (1.548, 2.764)		1.138 (0.811, 1.595)	
**eGFR ≥ 45 mL/min/1.73 m^2^**	Q1	19 (10.3)	Reference	0.108	Reference	0.143
	Q2	26 (14.4)	1.444 (0.799, 2.609)		2.191 (1.112, 4.316)	
	Q3	17 (9.6)	0.820 (0.426, 1.578)		1.045 (0.504, 2.163)	
	Q4	32 (18.3)	1.661 (0.941, 2.930)		1.988 (1.025, 3.855)	
	Q5	31 (26.1)	2.686 (1.517, 4.756)		2.336 (1.171, 4.663)	
**eGFR < 45 mL/min/1.73 m^2^**	Q1	85 (55.9)	Reference		Reference	
	Q2	85 (54.5)	1.198 (0.886, 1.619)		1.058 (0.736, 1.522)	
	Q3	96 (60.0)	1.162 (0.867, 1.557)		1.373 (0.986, 1.913)	
	Q4	93 (57.4)	1.234 (0.919, 1.658)		1.178 (0.842, 1.648)	
	Q5	148 (67.0)	1.602 (1.226, 2.095)		1.453 (1.061, 1.990)	
**Spot urine ACR < 300 mg/g**	Q1	35 (18.7)	Reference	0.053	Reference	<0.001
	Q2	39 (21.1)	1.144 (0.724, 1.805)		1.441 (0.825, 2.517)	
	Q3	30 (16.9)	0.790 (0.485, 1.287)		0.946 (0.529, 1.692)	
	Q4	22 (17.6)	0.902 (0.529, 1.539)		0.965 (0.530, 1.758)	
	Q5	35 (36.8)	2.393 (1.498, 3.824)		1.184 (0.659, 2.129)	
**Spot urine ACR ≥ 300 mg/g**	Q1	68 (45.6)	Reference		Reference	
	Q2	72 (47.7)	1.296 (0.930, 1.805)		1.005 (0.676, 1.493)	
	Q3	83 (52.2)	1.237 (0.898, 1.705)		1.731 (1.208, 2.480)	
	Q4	103 (48.8)	1.093 (0.805, 1.485)		1.262 (0.898, 1.774)	
	Q5	144 (58.8)	1.544 (1.157, 2.060)		1.361 (0.977, 1.895)	

The model was adjusted for age, sex, age-adjusted Charlson comorbidity index, primary renal disease, current smoking, medications (ACEi/ARBs, diuretics, number of antihypertensive drugs, statins), BMI, SBP, DBP, hemoglobin, albumin, fasting glucose, total cholesterol, LDL-C, HDL-C, TG, 25(OH) vitamin D, hs-CRP, serum creatinine and spot urine ACR. Abbreviations: ACR, albumin-to-creatinine ratio; CI, confidence interval; eGFR, estimated glomerular filtration rate; HR, hazard ratio; Q1, 1st quintile; Q2, 2nd quintile; Q3, 3rd quintile; Q4, 4th quintile; Q5, 5th quintile; UAGT/Cr, urinary angiotensinogen-to-creatinine ratio.

## Data Availability

Not applicable.

## Supplementary Materials

The following supporting information can be downloaded at: https://www.mdpi.com/article/10.3390/biom12091280/s1, Figure S1: Kaplan–Meier survival curve for cumulative incidence of decline in kidney function by UAGT/Cr; Figure S2: Kaplan–Meier survival curve for cumulative incidence of onset of ESRD by UAGT/Cr; Figure S3: Restricted cubic spline of UAGT/Cr on decline in kidney function; Figure S4: Restricted cubic spline of UAGT/Cr on onset of ESRD; Table S1: HRs for decline in kidney function by UAGT/Cr level; Table S2: HRs for onset of ESRD by UAGT/Cr level; Table S3: HRs for composite renal event by UAGT/Cr level after excluding the subjects at CKD stage 1; Table S4: HRs for composite renal event by UAGT/Cr level after excluding the subjects at CKD stage 5; Table S5: HRs for composite renal event by UAGT/Cr level after excluding the subjects with DM; Table S6: HRs for composite renal event by UAGT/Cr level after excluding the subjects with PKD; Table S7: HRs for composite renal event by UAGT/Cr level in the quartile division; Table S8: HRs for composite renal event by UAGT/Cr level, where the co-variate ‘serum creatinine level’ was replaced with ‘eGFR’.
