# Supplementary material for: Urinary Angiotensinogen and Progression of Chronic Kidney Disease: Results from KNOW-CKD Study
Source: Biomolecules. 2022 Sep 10;12(9):1280. doi: 10.3390/biom12091280 (PMC9496033; doi:10.3390/biom12091280)
Supplement: Supplementary file 1 [file biomolecules-12-01280-s001.zip › biomolecules-1880696-supplementary.pdf]

Supplemental Material

Urinary Angiotensinogen and Progression of Chronic Kidney Disease: Results from KNOW-CKD Study

Sang Heon Suh [1], Tae Ryom Oh [1], Hong Sang Choi [1], Eun Mi Yangi [2], Chang Seong Kim [1], Eun Hui Bae [1], Seong Kwon Ma [1], Kook-Hwan Oh [3], Ji Yong Jung [4], Young Youl Hyun [5] and Soo Wan Kim [1,*]

[1]Department of Internal Medicine, Chonnam National University Medical School and Chonnam National University Hospital, Gwangju 64169, Korea

[2]Department of Pediatrics, Chonnam National University Medical School and Chonnam National University Hospital, Gwangju 64169, Korea

[3]Department of Internal Medicine, Seoul National University Hospital, Seoul 03080, Korea

[4]Department of Internal Medicine, Gachon University of Medicine and Science, Incheon 21565, Korea

[5]Department of Internal Medicine, Kangbuk Samsung Hospital, Sungkyunkwan University School of Medicine, Seoul, 03181, Korea

Running title: UAGT and CKD progression

Corresponding authors

*Soo Wan Kim, M.D., Ph.D., Department of Internal Medicine, Chonnam National University Medical School, 42 Jebongro, Gwangju 61469, Korea, Tel: +82-62-220-6271, Fax: +82-62-225-8578, Email: skimw@chonnam.ac.kr

Table of contents

Figure S1. Kaplan-Meier survival curve for cumulative incidence of decline of kidney function by UAGT/Cr.

Figure S2. Kaplan-Meier survival curve for cumulative incidence of onset of ESRD by UAGT/Cr.

Figure S3. Restricted cubic spline of UAGT/Cr on decline of kidney function.

Figure S4. Restricted cubic spline of UAGT/Cr on onset of ESRD.

Table S1. HRs for decline of kidney function by UAGT/Cr level.

Table S2. HRs for onset of ESRD by UAGT/Cr level.

Table S3. HRs for composite renal event by UAGT/Cr level after excluding the subjects at CKD stage 1.

Table S4. HRs for composite renal event by UAGT/Cr level after excluding the subjects at CKD stage 5.

Table S5. HRs for composite renal event by UAGT/Cr level after excluding the subjects with DM.

Table S6. HRs for composite renal event by UAGT/Cr level after excluding the subjects with PKD.

Table S7. HRs for composite renal event by UAGT/Cr level in the quartile division.

Table S8. HRs for composite renal event by UAGT/Cr level, where the co-variate 'serum creatinine level' was replaced with 'eGFR'.

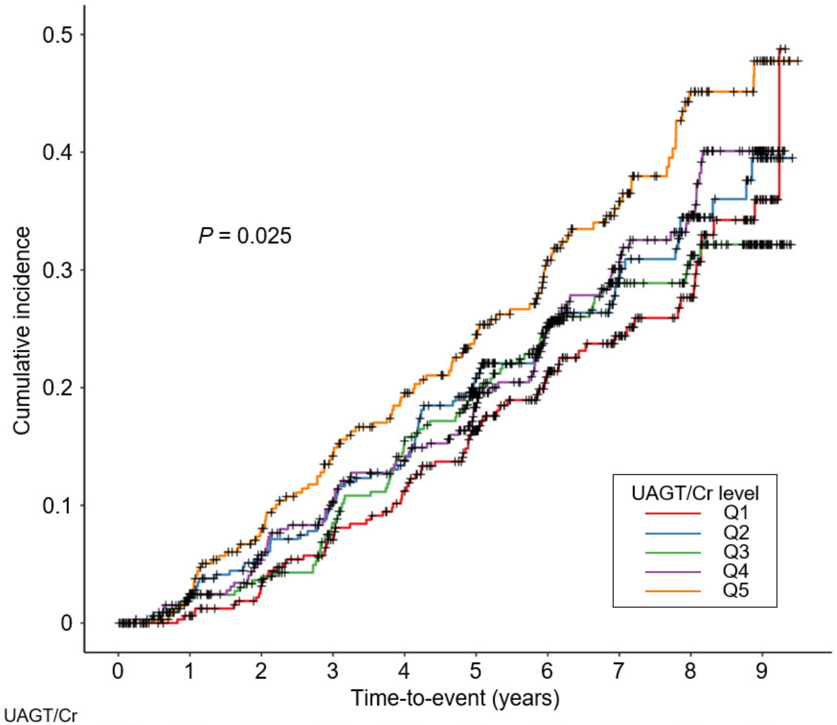

UAGT/Cr	0	1	2	3	4	5	6	7	8	9
Q1	337	323	304	276	254	214	155	113	78	29
Q2	337	306	278	257	240	196	142	93	64	25
Q3	337	322	306	279	253	216	162	112	84	33
Q4	337	314	289	262	244	204	152	118	88	31
Q5	340	308	274	245	222	190	145	105	64	34

Numbers at risk

Figure S1. Kaplan-Meier survival curve for cumulative incidence of decline of kidney function by UAGT/Cr.

Note: *P* value by Log-rank test. Abbreviations: Q1, 1st quintile; Q2, 2nd quintile, Q3, 3rd quintile; Q4, 4th quintile; Q5, 5th quintile; UAGT/Cr, urinary angiotensinogen-to-creatinine ratio.

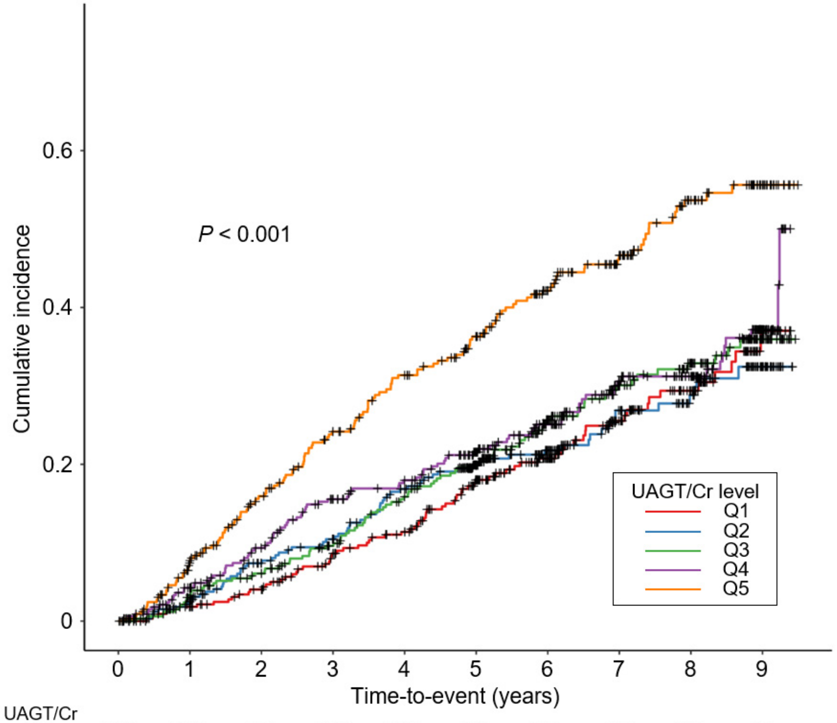

UAGT/Cr	0	1	2	3	4	5	6	7	8	9
Q1	337	319	302	273	254	209	151	111	75	24
Q2	337	306	274	256	230	192	137	92	68	27
Q3	337	319	300	277	253	216	162	112	85	31
Q4	337	309	281	252	236	200	151	117	93	35
Q5	340	291	250	216	190	162	127	92	56	23

Numbers at risk

Figure S2. Kaplan-Meier survival curve for cumulative incidence of onset of ESRD by UAGT/Cr.

Note: *P* value by Log-rank test. Abbreviations: Q1, 1st quintile; Q2, 2nd quintile, Q3, 3rd quintile; Q4, 4th quintile; Q5, 5th quintile; UAGT/Cr, urinary angiotensinogen-to-creatinine ratio.

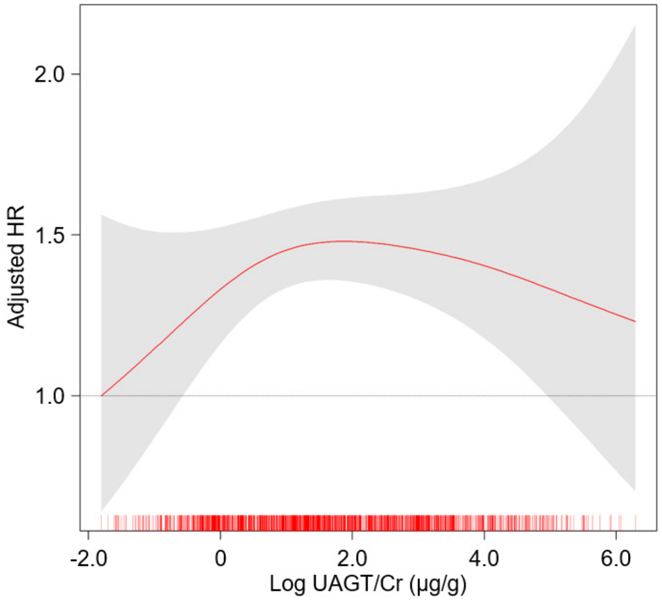

Figure S3. Restricted cubic spline of UAGT/Cr on decline of kidney function.

Note: Adjusted HR of UAGT/Cr as a continuous variable for composite renal event is depicted. The model was adjusted for age, sex, age-adjusted Charlson comorbidity index, primary renal disease, current smoking, medications (ACEi/ARBs, diuretics, number of antihypertensive drugs, statins), BMI, SBP, DBP, hemoglobin, albumin, fasting glucose, total cholesterol, LDL-C, HDL-C, TG, 25(OH) vitamin D, hs-CRP, serum creatinine and spot urine ACR.

Abbreviations: HR, hazard ratio; UAGT/Cr, urinary angiotensinogen-to-creatinine ratio.

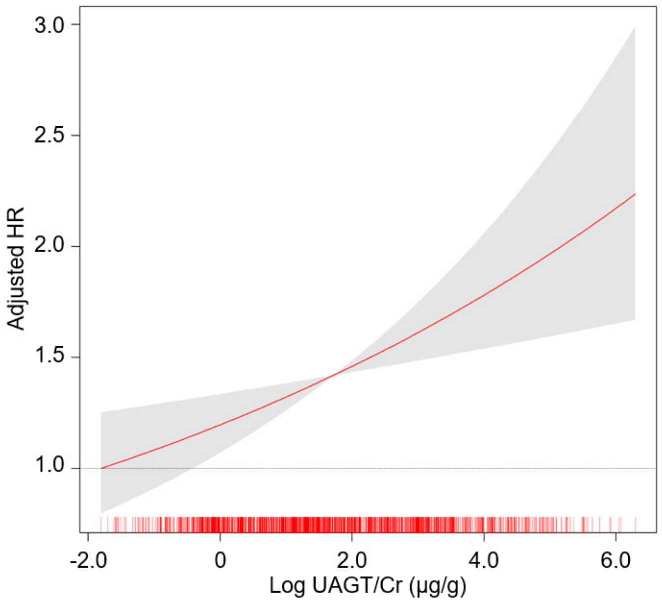

Figure S4. Restricted cubic spline of UAGT/Cr on onset of ESRD.

Note: Adjusted HR of UAGT/Cr as a continuous variable for composite renal event is depicted. The model was adjusted for age, sex, age-adjusted Charlson comorbidity index, primary renal disease, current smoking, medications (ACEi/ARBs, diuretics, number of antihypertensive drugs, statins), BMI, SBP, DBP, hemoglobin, albumin, fasting glucose, total cholesterol, LDL-C, HDL-C, TG, 25(OH) vitamin D, hs-CRP, serum creatinine and spot urine ACR.

Abbreviations: HR, hazard ratio; UAGT/Cr, urinary angiotensinogen-to-creatinine ratio.

Table S1. HRs for decline of kidney function by UAGT/Cr level.

	UAGT/Cr	Events, n (%)	Model 1		Model 2		Model 3		Model 4	
			HR (95%CIs)	*P* value	HR (95%CIs)	*P* value	HR (95%CIs)	*P* value	HR (95%CIs)	*P* value
Decline of kidney function	Q1	77 (22.8)	Reference		Reference		Reference		Reference	
	Q2	84 (24.9)	1.177 (0.844, 1.641)	0.338	1.384 (1.013, 1.891)	0.414	1.391 (0.991, 1.954)	0.056	1.402 (0.997, 1.972)	0.052
	Q3	84 (24.9)	1.060 (0.764, 1.473)	0.726	1.278 (0.931, 1.753)	0.129	1.309 (0.935, 1.832)	0.117	1.317 (0.939, 1.848)	0.111
	Q4	91 (27.0)	1.345 (0.978, 1.848)	0.068	1.245 (0.913, 1.699)	0.166	1.313 (0.943, 1.827)	0.106	1.284 (0.918, 1.795)	0.144
	Q5	108 (31.8)	1.632 (1.195, 2.228)	0.002	1.375 (1.019, 1.856)	0.037	1.309 (0.945, 1.814)	0.105	1.204 (0.858, 1.691)	0.283

Note: Model 1, unadjusted model. Model 2, model 1 + adjusted for age, sex, age-adjusted Charlson comorbidity index, primary renal disease, current smoking, medication (ACEi/ARBs, diuretics, number of antihypertensive drugs, statins), BMI, SBP and DBP. Model 3, model 2 + adjusted for hemoglobin, albumin, total cholesterol, LDL-C, HDL-C, TG, fasting glucose, 25(OH) vitamin D, and, hs-CRP. Model 4, model 3 + serum creatinine and spot urine ACR. Abbreviations: CI, confidence interval; HR, hazard ratio; Q1, 1st quintile; Q2, 2nd quintile, Q3, 3rd quintile; Q4, 4th quintile; Q5, 5th quintile; UAGT/Cr, urinary angiotensinogen-to-creatinine ratio.

Table S2. HRs for onset of ESRD by UAGT/Cr level.

	UAGT/Cr	Events, n (%)	Model 1		Model 2		Model 3		Model 4	
			HR (95%CIs)	*P* value	HR (95%CIs)	*P* value	HR (95%CIs)	*P* value	HR (95%CIs)	*P* value
Onset of ESRD	Q1	77 (22.8)	Reference		Reference		Reference		Reference	
	Q2	74 (22.0)	1.003 (0.712, 1.413)	0.987	1.208 (0.875, 1.668)	0.250	1.256 (0.887, 1.780)	0.199	0.831 (0.565, 1.224)	0.349
	Q3	91 (27.0)	1.152 (0.837, 1.587)	0.385	1.574 (1.153, 2.148)	0.004	1.562 (1.120, 2.178)	0.009	1.470 (1.054, 2.049)	0.023
	Q4	94 (27.9)	1.368 (0.995, 1.866)	0.053	1.480 (1.008, 2.013)	0.013	1.568 (1.133, 2.171)	0.007	1.336 (0.963, 1.852)	0.083
	Q5	142 (41.8)	2.282 (1.700, 3.063)	< 0.001	2.006 (1.508, 2.668)	< 0.001	1.901 (1.390, 2.600)	< 0.001	1.444 (1.050, 1.987)	0.024

Note: Model 1, unadjusted model. Model 2, model 1 + adjusted for age, sex, age-adjusted Charlson comorbidity index, primary renal disease, current smoking, medication (ACEi/ARBs, diuretics, number of antihypertensive drugs, statins), BMI, SBP and DBP. Model 3, model 2 + adjusted for hemoglobin, albumin, total cholesterol, LDL-C, HDL-C, TG, fasting glucose, 25(OH) vitamin D, and, hs-CRP. Model 4, model 3 + serum creatinine and spot urine ACR. Abbreviations: CI, confidence interval; ESRD, end-stage renal disease; HR, hazard ratio; Q1, 1st quintile; Q2, 2nd quintile, Q3, 3rd quintile; Q4, 4th quintile; Q5, 5th quintile; UAGT/Cr, urinary angiotensinogen-to-creatinine ratio.

Table S3. HRs for composite renal event by UAGT/Cr level after excluding the subjects at CKD stage 1.

	UAGT/Cr	Events, n (%)	Model 1		Model 2		Model 3		Model 4	
			HR (95%CIs)	*P* value	HR (95%CIs)	*P* value	HR (95%CIs)	*P* value	HR (95%CIs)	*P* value
Composite renal event	Q1	101 (36.1)	Reference		Reference		Reference		Reference	
	Q2	107 (39.5)	1.277 (0.952, 1.712)	0.103	1.444 (1.070, 1.948)	0.016	1.465 (1.112, 1.929)	0.007	1.152 (0.837, 1.586)	0.385
	Q3	111 (42.4)	1.256 (0.944, 1.673)	0.118	1.440 (1.071, 1.936)	0.016	1.453 (1.010, 1.918)	0.008	1.403 (1.043, 1.888)	0.025
	Q4	120 (44.3)	1.495 (1.132, 1.975)	0.005	1.508 (1.127, 2.016)	0.006	1.487 (1.132, 1.952)	0.004	1.289 (0.961, 1.730)	0.090
	Q5	175 (56.8)	2.225 (1.713, 2.892)	< 0.001	1.804 (1.361, 2.390)	< 0.001	1.966 (1.526, 2.533)	< 0.001	1.449 (1.087, 1.932)	0.012

Note: Model 1, unadjusted model. Model 2, model 1 + adjusted for age, sex, age-adjusted Charlson comorbidity index, primary renal disease, current smoking, medication (ACEi/ARBs, diuretics, number of antihypertensive drugs, statins), BMI, SBP and DBP. Model 3, model 2 + adjusted for hemoglobin, albumin, total cholesterol, LDL-C, HDL-C, TG, fasting glucose, 25(OH) vitamin D, and, hs-CRP. Model 4, model 3 + serum creatinine and spot urine ACR. Abbreviations: CI, confidence interval; HR, hazard ratio; Q1, 1st quintile; Q2, 2nd quintile, Q3, 3rd quintile; Q4, 4th quintile; Q5, 5th quintile; UAGT/Cr, urinary angiotensinogen-to-creatinine ratio.

Table S4. HRs for composite renal event by UAGT/Cr level after excluding the subjects at CKD stage 5.

	UAGT/Cr	Events, n (%)	Model 1		Model 2		Model 3		Model 4	
			HR (95%CIs)	*P* value	HR (95%CIs)	*P* value	HR (95%CIs)	*P* value	HR (95%CIs)	*P* value
Composite renal event	Q1	96 (29.4)	Reference		Reference		Reference		Reference	
	Q2	102 (31.5)	1.204 (0.891, 1.627)	0.226	1.440 (1.086, 1.910)	0.011	1.564 (1.150, 2.128)	0.004	1.397 (1.204, 1.907)	0.035
	Q3	100 (30.9)	1.063 (0.781, 1.419)	0.734	1.346 (1.009, 1.795)	0.042	1.450 (1.064, 1.975)	0.019	1.241 (0.906, 1.698)	0.179
	Q4	110 (34.9)	1.389 (1.041, 1.855)	0.026	1.458 (1.101, 1.931)	0.009	1.653 (1.223, 2.233)	0.001	1.288 (0.944, 1.756)	0.110
	Q5	143 (48.0)	2.164 (1.640, 2.854)	< 0.001	1.868 (1.433, 2.434)	< 0.001	1.861 (1.389, 2.495)	< 0.001	1.444 (1.069, 1.951)	0.017

Note: Model 1, unadjusted model. Model 2, model 1 + adjusted for age, sex, age-adjusted Charlson comorbidity index, primary renal disease, current smoking, medication (ACEi/ARBs, diuretics, number of antihypertensive drugs, statins), BMI, SBP and DBP. Model 3, model 2 + adjusted for hemoglobin, albumin, total cholesterol, LDL-C, HDL-C, TG, fasting glucose, 25(OH) vitamin D, and, hs-CRP. Model 4, model 3 + serum creatinine and spot urine ACR. Abbreviations: CI, confidence interval; HR, hazard ratio; Q1, 1st quintile; Q2, 2nd quintile, Q3, 3rd quintile; Q4, 4th quintile; Q5, 5th quintile; UAGT/Cr, urinary angiotensinogen-to-creatinine ratio.

Table S5. HRs for composite renal event by UAGT/Cr level after excluding the subjects with DM.

	UAGT/Cr	Events, n (%)	Model 1		Model 2		Model 3		Model 4	
			HR (95%CIs)	*P* value	HR (95%CIs)	*P* value	HR (95%CIs)	*P* value	HR (95%CIs)	*P* value
Composite renal event	Q1	62 (25.2)	Reference		Reference		Reference		Reference	
	Q2	69 (25.9)	1.136 (0.785, 1.644)	0.498	1.433 (1.009, 2.034)	0.044	1.515 (1.033, 2.224)	0.034	1.164 (0.773, 1.753)	0.466
	Q3	79 (29.5)	1.222 (0.860, 1.735)	0.264	1.429 (1.015, 2.012)	0.040	1.575 (1.093, 2.269)	0.015	1.515 (1.052, 2.182)	0.026
	Q4	81 (30.2)	1.364 (0.963, 1.931)	0.080	1.221 (0.870, 1.716)	0.249	1.464 (1.019, 2.104)	0.039	1.240 (0.860, 1.786)	0.249
	Q5	111 (48.9)	2.526 (1.814, 3.518)	< 0.001	2.117 (1.158, 2.913)	< 0.001	2.265 (1.602, 3.204)	< 0.001	1.901 (1.335, 2.707)	< 0.001

Note: Model 1, unadjusted model. Model 2, model 1 + adjusted for age, sex, age-adjusted Charlson comorbidity index, primary renal disease, current smoking, medication (ACEi/ARBs, diuretics, number of antihypertensive drugs, statins), BMI, SBP and DBP. Model 3, model 2 + adjusted for hemoglobin, albumin, total cholesterol, LDL-C, HDL-C, TG, fasting glucose, 25(OH) vitamin D, and, hs-CRP. Model 4, model 3 + serum creatinine and spot urine ACR. Abbreviations: CI, confidence interval; HR, hazard ratio; Q1, 1st quintile; Q2, 2nd quintile, Q3, 3rd quintile; Q4, 4th quintile; Q5, 5th quintile; UAGT/Cr, urinary angiotensinogen-to-creatinine ratio.

Table S6. HRs for composite renal event by UAGT/Cr level after excluding the subjects with PKD.

	UAGT/Cr	Events, n (%)	Model 1		Model 2		Model 3		Model 4	
			HR (95%CIs)	*P* value	HR (95%CIs)	*P* value	HR (95%CIs)	*P* value	HR (95%CIs)	*P* value
Composite renal event	Q1	90 (31.2)	Reference		Reference		Reference		Reference	
	Q2	97 (37.2)	1.358 (0.996, 1.852)	0.053	1.483 (1.084, 2.028)	0.014	1.519 (1.106, 2.086)	0.010	1.160 (0.829, 1.625)	0.386
	Q3	94 (35.7)	1.182 (0.868, 1.608)	0.289	1.489 (1.088, 2.038)	0.013	1.456 (1.059, 2.003)	0.021	1.360 (0.989, 1.871)	0.058
	Q4	108 (38.4)	1.424 (1.060, 1.913)	0.019	1.623 (1.200, 2.196)	0.002	1.576 (1.159, 2.141)	0.004	1.257 (0.923, 1.713)	0.147
	Q5	155 (51.8)	2.179 (1.650, 2.877)	< 0.001	2.125 (1.595, 2.831)	< 0.001	1.844 (1.374, 2.475)	< 0.001	1.363 (1.009, 1.841)	0.044

Note: Model 1, unadjusted model. Model 2, model 1 + adjusted for age, sex, age-adjusted Charlson comorbidity index, primary renal disease, current smoking, medication (ACEi/ARBs, diuretics, number of antihypertensive drugs, statins), BMI, SBP and DBP. Model 3, model 2 + adjusted for hemoglobin, albumin, total cholesterol, LDL-C, HDL-C, TG, fasting glucose, 25(OH) vitamin D, and, hs-CRP. Model 4, model 3 + serum creatinine and spot urine ACR. Abbreviations: CI, confidence interval; HR, hazard ratio; Q1, 1st quintile; Q2, 2nd quintile, Q3, 3rd quintile; Q4, 4th quintile; Q5, 5th quintile; UAGT/Cr, urinary angiotensinogen-to-creatinine ratio.

Table S7. HRs for composite renal event by UAGT/Cr level in the quartile division.

	UAGT/Cr	Events, n (%)	Model 1		Model 2		Model 3		Model 4	
			HR (95%CIs)	*P* value	HR (95%CIs)	*P* value	HR (95%CIs)	*P* value	HR (95%CIs)	*P* value
Composite renal event	1st quarter	132 (31.3)	Reference		Reference		Reference		Reference	
	2nd quarter	152 (36.0)	1.187 (0.924, 1.524)	0.179	1.381 (1.089, 1.751)	0.008	1.325 (1.026, 1.711)	0.031	1.111 (0.851, 1.449)	0.440
	3rd quarter	136 (32.2)	1.072 (0.834, 1.377)	0.589	1.247 (0.976, 1.594)	0.078	1.337 (1.030, 1.734)	0.029	1.148 (0.883, 1.491)	0.303
	4th quarter	212 (50.2)	2.050 (1.629, 2.580)	< 0.001	1.781 (1.424, 2.228)	< 0.001	1.758 (1.378, 2.244)	< 0.001	1.385 (1.078, 1.778)	0.011

Note: Model 1, unadjusted model. Model 2, model 1 + adjusted for age, sex, age-adjusted Charlson comorbidity index, primary renal disease, current smoking, medication (ACEi/ARBs, diuretics, number of antihypertensive drugs, statins), BMI, SBP and DBP. Model 3, model 2 + adjusted for hemoglobin, albumin, total cholesterol, LDL-C, HDL-C, TG, fasting glucose, 25(OH) vitamin D, and, hs-CRP. Model 4, model 3 + serum creatinine and spot urine ACR. Abbreviations: CI, confidence interval; HR, hazard ratio; UAGT/Cr, urinary angiotensinogen-to-creatinine ratio.

Table S8. HRs for composite renal event by UAGT/Cr level, where the co-variate 'serum creatinine level' was replaced with 'eGFR'.

	UAGT/Cr	Adjusted	
		HR (95%CIs)	*P* value
Composite renal event	Q1	Reference	
	Q2	1.278 (0.948, 1.724)	0.108
	Q3	1.286 (0.958, 1.726)	0.095
	Q4	1.197 (0.892, 1.604)	0.230
	Q5	1.427 (1.078, 1.888)	0.013

Note: Adjusted for age, sex, age-adjusted Charlson comorbidity index, primary renal disease, current smoking, medication (ACEi/ARBs, diuretics, number of antihypertensive drugs, statins), BMI, SBP, DBP, hemoglobin, albumin, total cholesterol, LDL-C, HDL-C, TG, fasting glucose, 25(OH) vitamin D, hs-CRP, eGFR and spot urine ACR. Abbreviations: CI, confidence interval; HR, hazard ratio; Q1, 1st quintile; Q2, 2nd quintile, Q3, 3rd quintile; Q4, 4th quintile; Q5, 5th quintile; UAGT/Cr, urinary angiotensinogen-to-creatinine ratio.
